# COVID-19 and the rising scourge of antimicrobial resistance: A perspective from Pakistan

**DOI:** 10.1016/j.amsu.2022.104262

**Published:** 2022-07-31

**Authors:** Fatima Faraz, Mohammad Ebad Ur Rehman, Aiza Iqbal, Ayesha Azeem

**Affiliations:** Department of Medicine, Rawalpindi Medical University, Block E Satellite Town, Rawalpindi, Pakistan; Department of Surgery, Rawalpindi Medical University, Block E Satellite Town, Rawalpindi, Pakistan

**Keywords:** COVID-19, Antimicrobial resistance, Antimicrobial stewardship, AMR, Antimicrobial Resistance

To The Editor,

The World Health Organization has recognized antimicrobial resistance as one of the most critical public health threats facing humanity, with more than 5 million deaths being attributed to drug-resistant infections in 2019, according to a systematic analysis of AMR data from more than 200 countries [1]. Although AMR affects almost all countries, low and middle-income countries bear the brunt of this ‘silent pandemic’ due to their limited resources, poor antimicrobial stewardship and rising pollution [2]. In 2019, the South Asian region was second only to Sub-Saharan Africa in deaths associated with or attributable to AMR [1].

The COVID-19 pandemic has presented the most unprecedented challenge to public health since the era of the influenza pandemic of 1918, having far-reaching impact on the physical, social, psychological and mental health of the population, in addition to bringing global economy and trade to a standstill [3]. With its ruinous effects on all the major sectors and industries, the governments were forced to impose quarantines, isolation, border-shutdowns and restrictions on travel and flights [4]. Despite all these efforts, the incidence of COVID continued to rise at a tremendous rate between 2019 and 2021 [5]. One potential long-term consequence of the pandemic that has gone largely unnoticed by healthcare professionals and policy-makers is the rise in AMR due to the imprudent and unwarranted use of antibiotics.

The administration of antibiotic therapy in COVID-19 patients is disproportionately greater than the reported incidence of bacterial co-infection. Pooled results from a meta-analysis demonstrated that bacterial co-infection and secondary infection are reported in only 3.5% and 14.3% of COVID-19 patients respectively, but 71.9% of patients received antibiotic therapy [6]. Excessive use of antibiotics has fast-tracked the loss of efficacy of routinely used drug groups like carbapenems as well as second-line options such as colistin.

In contrast, earlier studies conducted in China suggested that up to half (50%) of patients who died of Covid-19 in hospitals had bacterial co-infections [7]. These results may have contributed to the excessive use by empirical antibiotic therapy in COVID-19. Prior to the pandemic, most global health organisations were investing resources in Antimicrobial Stewardship Programmes. However, the pandemic fuelled the excessive use of antibiotics, leading to a drastic increase in AMR [8].

With an ever-growing list of highly resistant pathogens, Pakistan is not exempt from the insidious threat of AMR [9]. Efforts have been made to highlight and curtail resistance by promoting judicious use of antibiotics; a National Action Plan to combat AMR was devised in 2018 [10]. However, definite progress has been hindered by limited awareness among the public as well as the policy makers, widespread use of antibiotics in agriculture and cattle-rearing, polypharmacy and limited application of resources to combat AMR [11]. Several studies have investigated the rising trend of AMR in Pakistan. Antibiotics were frequently used as frontline therapy against the virus. The most commonly prescribed therapy for COVID-19 was azithromycin, most likely due to its anti-inflammatory action. It was discovered that most prescribers did not take the severity of disease into account when dispensing antibiotics for resistant infections, thus exacerbating AMR and risk of complications [12,13]. According to the results of a cross sectional study, fewer than one-fourth of prescribers (n = 91, 23.5%) based their choice of prescription on local AMR data [14]. Some authors have hypothesized that the lack of established antiviral protocols to combat COVID-19 causes healthcare professionals to resort to prescribing antibiotics [15].

However, there may be cases in which timely initiation of empirical antibiotic therapy is justified. The Dutch Working Party on Antibiotic Policy has recommended that a physician should strongly consider antibiotics in patients with laboratory and radiological findings consistent with bacterial co-infection. In addition, empirical antibiotic therapy may be crucial in immunodeficiency patients awaiting lab results [16]. All in all, they should be administered when secondary bacterial infections are suspected or proved.

Although a growing body of evidence suggests antibiotics are inefficacious and unnecessary in COVID-19 infections, further research is ongoing and will elucidate this matter. The treatment of COVID-19 must be based on evidence and physicians must adopt a multi-factorial approach rather than administering medicines that have unknown efficacy [17]. Nevertheless, the pandemic has made planning and implementation of programs to mitigate the rising AMR in Pakistan even more crucial. The threat of AMR has remained largely inconspicuous in the public eye; awareness campaigns are thus a critical component in the fight against AMR. Early detection of novel resistant strains, establishment of resilient antibiotic stewardship programs and allocation of resources towards the development of new drugs in the clinical pipeline have become necessary steps in combating AMR. Collaboration between all stakeholders is vital in making concrete progress towards preventing an era of untreatable diseases.

## Ethical approval

Not applicable.

## Sources of funding

None.

## Author contribution

Fatima Faraz: Study conception, write-up, critical review and approval of the final version, Mohammad Ebad Ur Rehman: Study conception, write-up, critical review and approval of the final version, Aiza Iqbal: Study conception, critical review and approval of the final version, Ayesha Azeem: Study conception, critical review and approval of the final version.

## Registration of research studies

**1.N**ame of the registry: Not applicable.

2.Unique Identifying number or registration ID: Not applicable.

3.Hyperlink to your specific registration (must be publicly accessible and will be checked): Not **applicable**.

## Guarantor

Fatima Faraz.

Mohammad Ebad Ur Rehman.

## Declaration of competing interest

The authors have no conflicts of interest.
